# A Comparative Study of Three Measurement Methods of Chinese Character Recognition for L2 Chinese Learners

**DOI:** 10.3389/fpsyg.2022.753913

**Published:** 2022-03-25

**Authors:** Haiwei Zhang, Sun-A. Kim, Xueyan Zhang

**Affiliations:** ^1^School of Chinese as a Second Language, Peking University, Beijing, China; ^2^College of International Education, Minzu University of China, Beijing, China; ^3^Department of Chinese and Bilingual Studies, Hong Kong Polytechnic University, Kowloon, Hong Kong SAR, China

**Keywords:** Chinese characters, character recognition, character test, Chinese as a second language (CSL) reading, Chinese reading acquisition

## Abstract

Measuring Chinese character recognition ability is essential in research on character learning among learners of Chinese as a second language (CSL). Three methods are typically used to evaluate character recognition competence by investigating the following properties of a given character: (a) pronunciation (phonological method), (b) meaning (semantic method), and (c) pronunciation and meaning (phonological and semantic or PS method). However, no study has explored the similar or dissimilar outcomes that these three measurements might yield. The current study examined this issue by testing 162 CSL learners with various L1 backgrounds and Chinese proficiency levels. Participants' performance in character recognition measured using a phonological method, a semantic method, and a PS method was compared, which led to two major findings. In terms of similarity, participants' performance in character recognition and the influence of L1 background and Chinese proficiency level on character recognition was similar across the three methods. As for differences, the semantic method could yield a character recognition test with better quality than the other two methods, and the three methods yielded different best fitting models and showed different predictions for Chinese proficiency across different L1 groups. Theoretical and practical implications of these findings are proposed.

## Introduction

The Chinese script is categorized as a logographic or morphosyllabic writing system (DeFrancis, 1984). In general, although there are some mono-morphemic two-character words (e.g., 蝴蝶 húdié “butterfly,” 玻璃 bōli “glass”), a majority of Chinese characters carry a certain meaning as a morpheme and can be combined with another character to form a new word. For instance, 手机 (shǒujī, “mobile phone”) is composed of 手 (shǒu, “hand”) and 机 (jī, “machine”), with each character representing a morpheme. Chinese characters are recognized as the basic units of Chinese words and sentences, so sufficient Chinese character knowledge is fundamental for reading and writing skills for both native Chinese speakers and learners of Chinese as a second language (CSL). Recognizing a Chinese character generally means decoding both its pronunciation (phonology) and meaning (semantics), yet the sublexical or syntactic knowledge of the target character could also be activated during character recognition (Tsai et al., 2004; Yan et al., 2009, 2012; Tsang et al., 2017; Yeh et al., 2017; Pan et al., 2019).

Based on the number of orthographic components within a character, Chinese characters are classified as simple characters or compound characters. A simple character is composed of a single undividable component, such as 木 (mù, “wood”), while a compound character is comprised of two or more components, as in 森 (sēn, “forest” with three 木 glyphs). It is estimated that simple characters and compound characters make up about 15 and 85% respectively, of commonly used modern characters in mainland China (Shu et al., 2003). Decoding the pronunciation and meaning of Chinese characters is not an easy task due to the opaque mapping between orthography (visual forms) and phonology, and between orthography and semantics.

The relationship between orthography and phonology in a character is opaque, which poses considerable difficulty in extracting character pronunciation from written forms. Some semantic-phonetic compound characters, which make up about 70% of commonly used characters (Shu et al., 2003), contain a component bearing phonological information related to the pronunciation of the entire character, which is called a *phonetic radical*. However, the chance that a phonetic radical accurately indicates the character's exact pronunciation is low. According to the degree to which a phonetic radical corresponds to a whole character's pronunciation, phonetic compound characters can be grouped into three types. The first type is regular compound characters, in which a phonetic radical and a whole character share the same syllable, without considering tones, such as 请 (q$\bar{ı}$ng, “invite”) and 青 (q$\bar{ı}$ng, “green”). The second type is semi-regular compound characters, where a phonetic radical and a whole character share the same onset or rime, such as 忙 (máng, “busy”) and 亡 (wáng, “dead”), and 倩 (qiàn, “pretty”) and 青 (q$\bar{ı}$ng, “green”). The third type is irregular compounds: the pronunciations of a phonetic radical and a whole character are entirely different, as seen in 冯 (féng, “a surname”) and 马 (mǎ, “horse”). However, only about 35% of commonly used compound characters are regular (Li et al., 1992; Shu et al., 2003; Wan, 2005). Even so, native Chinese speakers (Tzeng et al., 1995; Chan and Nunes, 1998; Zhou and Marslen-Wilson, 1999a,b,c; Anderson et al., 2003; Ho et al., 2003; He et al., 2005; Lee et al., 2005; Cai et al., 2012; Yin and McBride, 2015; Tong et al., 2017; Li et al., 2018, 2020) and CSL learners (Williams, 2013; Tong and Yip, 2014; Wei et al., 2014; Tong et al., 2015; Liu et al., 2020) still rely on the phonetic radicals to extract character pronunciation.

Although Chinese characters are considered logographic or meaning-based, decoding the exact meaning of characters is also difficult. In the course of being used for thousands of years, the visual forms of Chinese characters have undergone change, and the meaning of most Chinese characters cannot be immediately inferred from their orthographic appearance. For instance, the simple character 目 (mù, “eye”) was written in the shape of an eye, 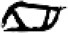 or 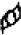, about 3,000 years ago when these characters were written on oracle bones, but the current rectangular form has lost its resemblance to an eye. Most compound characters contain a semantic component, called a *semantic radical*, but a semantic radical roughly indicates an approximate semantic category to which a character belongs. In the character 枫 (fēng, “maple tree”) for example, the semantic radical 木 (mù, “tree”) only suggests that 枫 may be semantically related to trees. Most characters containing the semantic radical 木, such as 村 (cūn, “village”), 杏 (xìng, “apricot”), 柏 (bǎi, “cypress”), or 框 (kuāng, “frame”), are associated with trees to some extent, but correctly guessing the exact meaning of a character in modern Chinese based on its semantic radical is difficult. However, Chinese children still use semantic radicals to derive character meaning in studies involving Pinyin-character mapping (Shu and Anderson, 1997), generating names for novel objects (Chan and Nunes, 1998), semantic category judgements (Ho et al., 1999), priming experiments (Zou et al., 2019), and eye-tracking experiments (Li et al., 2019). Some studies further found that the effect of semantic radicals in retrieving character meaning might interact with imageability and neighborhood density (Feldman and Siok, 1999; Li et al., 2020). Similarly, CSL learners rely on semantic radicals to extract unfamiliar character meaning (Taft and Chung, 1999; Lü et al., 2014; Nguyen et al., 2017) or to complete semantic categorization tasks (Williams, 2013) and lexical decision tasks (Williams and Bever, 2010), and their productive knowledge of semantic radicals uniquely predicted their performance in word reading (Su and Kim, 2014).

Based on these components of Chinese character recognition, three methods have been commonly utilized by researchers to measure Chinese learners' character recognition. The first method focuses on learners' performance in character pronunciation, that is, the phonological method^1^. The second method emphasizes learners' performance on character meaning, or the semantic method. The third method, known as the phonological and semantic (PS) method, concentrates on learners' performance in both character pronunciation and character meaning. Both similar and dissimilar results have been generated from these different methods; however, researchers have not reached a consensus about which method is optimal in measuring Chinese character recognition skills. This is an important practical issue for researchers studying native Chinese speakers and CSL learners because measuring character recognition skill is the basis for carrying out research on Chinese literacy skills. Considering the increasing importance of CSL learning (Ma et al., 2017; Gong et al., 2018, 2020a,b) and growing attention to the acquisition of characters by CSL learners (Li, 2020), such study is significant for theories concerning character acquisition and classroom instruction.

## Literature Review

### Measuring Chinese Character Recognition

Different models have been proposed for visual word recognition, such as the interactive-activation model (McClelland and Rumelhart, 1981), the dual-route cascaded model (Coltheart et al., 2001), the distributed representation triangle model (Plaut and Booth, 2000), and the lexical constituency model (LCM; Perfetti et al., 2005). Although these models hold different assumptions about the mechanisms underlying word recognition, a consensus has been reached about the interaction between orthographic, phonological, and semantic representations (Seidenberg and McClelland, 1989; Lupker, 2005), which can be also observed in models exploring Chinese character recognition for CSL learners (Tong et al., 2015) and native Chinese speakers (Yang et al., 2006, 2009; Chang et al., 2016b; Reichle and Yu, 2018). Of these models, the LCM has been well validated across alphabetic (e.g., English) and nonalphabetic (e.g., Chinese) writing systems (Perfetti and Liu, 2006). LCM assumes that a word representation consists of three interlinked constituents: orthography, phonology, and semantics, and “written word identification entails the retrieval of a phonological form and meaning information from a graphic form” (Perfetti et al., 2005, p. 46).

For evaluating participants' character recognition skills, researchers have measured these from three perspectives: phonology, semantics, and phonology plus semantics. There are three commonly used phonological methods. The first method is requiring participants to provide the pronunciation of a Chinese character by reading the character aloud, and this has been widely used with native Chinese speakers across the mainland China, Hong Kong, and Taiwan regions (Huang and Hanley, 1995; Ho and Bryant, 1997; McBride and Kail, 2002; McBride-Chang et al., 2003; Shu et al., 2008; Tong et al., 2009, 2011; Pan et al., 2011; Li et al., 2012; Wei et al., 2014), and with CSL learners (Wu et al., 2017). The second is asking the participants to write down the character pronunciation in Pinyin (the official romanization system for Chinese in mainland China). This method has been commonly observed in studies of CSL learners (Everson, 1998; Tseng et al., 2016; Gao, 2017; Hao, 2018). The third method is requiring participants to indicate character pronunciation using Zhuyin Fuhao (the official transliteration system for Chinese in Taiwan) and has been used mainly for Chinese children (Liao et al., 2008; Liao and Kuo, 2011) and CSL learners (Tseng et al., 2016) in Taiwan. Phonological methods are usually developed in-house and have not been standardized or validated, but some researchers have used a standardized test for character pronunciations, such as the *Graded Chinese Character Recognition Test* in Taiwan (Huang, 2001) and *The Hong Kong Test of Specific Learning Disabilities in Reading and Writing (HKT-SpLD)* (Ho et al., 2000).

The semantic method asks participants to provide the meaning of a character. Requiring Chinese-speaking children to form words or phrases using a target character (Wang and Tao, 1996) or asking CSL learners to translate characters into their L1 (Ke, 1996; Everson, 1998; Jiang, 2003) are some examples of semantic tasks. Semantic methods are used by some researchers to measure how many characters a person can recognize, and one particular character recognition test developed by Wang and Tao (1996) has been widely used in China.

A PS method elicits both the pronunciation and the meaning of a character. This type is mainly used in research exploring character recognition ability among Chinese-speaking children (Hung et al., 2008; Wen et al., 2015) or CSL learners (Ke, 1996; Jiang et al., 2006; Zhang et al., 2021).

### Features of Three Measurement Methods of Character Recognition

Phonological methods have been popular for the following reasons. A majority of previous studies on Chinese character recognition have focused on character naming, so a phonological measurement would be ideal for such research. Also, phonological methods save time and effort, as it generally takes less than 5 min to read out 100 characters for Chinese children with normal cognitive development. Moreover, phonological methods are closely correlated with semantic methods requiring much more time, for both native Chinese speakers (Perfetti and Zhang, 1995; Perfetti and Tan, 1998; Myers et al., 2007) and CSL learners, in particular those who are from the non-Sinographosphere^2^ (Everson, 1998; Jiang, 2003). Evidence from neuropsychological studies have also shown that phonological and semantic processing of Chinese characters overlap in the left middle frontal gyrus, the left superior parietal lobule, and the left mid-fusiform gyrus (Wu et al., 2012). Therefore, considering the limited time and funding available to most researchers, phonological methods could be optimal for collecting data on character recognition.

In spite of the correlation and the overlaps between phonological methods and semantic methods, these two methods are distinct in several respects. They differ in the cognitive processes involved. Generally, accessing character pronunciation activates phonological representations of written characters, but questions eliciting meaning depend on the activation of semantic representations from orthographic and/or phonological features. Researchers have argued for the importance of phonological activation in semantic processing using different tasks, such as masked priming experiments (Tan et al., 1996), primed-naming experiments (Perfetti and Tan, 1998), and semantic judgement (Perfetti and Zhang, 1995). However, researchers have also found that the mediation of phonology in accessing a character's meaning might not be obligatory in Chinese speakers with normal cognitive skills in priming experiments (Zhou et al., 1999; Chou, 2000; Wu and Chen, 2000; Zhou and Marslen-Wilson, 2000; Chen and Shu, 2001) and eye-tracking paradigms (Tsai et al., 2012; Pan et al., 2016, 2021). Similar results were observed among Chinese-speaking aphasic patients (Han and Bi, 2009) and Kanji recognition in Japanese (Wydell et al., 1993; Sakuma et al., 1998; Chen et al., 2007), where phonological contribution to the activation of kanji meaning were found to be condition-specific. In addition, brain areas with separate activation for the phonological processing of characters (such as the posterior dorsal region of the inferior/middle frontal gyrus) and for semantic processing (such as the anterior ventral region of the middle frontal gyrus) have been reported (Booth et al., 2006; Wu et al., 2012). That is, phonological and semantic processing of characters might have specific, separate pathways to some extent.

Phonological methods might be easier than semantic methods in measuring character recognition. The first reason relates to the nature of phonological and semantic clues in Chinese characters, since, as mentioned, accessing the pronunciation and meaning of a simple character is difficult due to the insufficient phonological and semantic cues within the character. For a compound character, Shu et al. (2003) concluded that the effect size of a phonetic component on character reading (Ho and Bryant, 1997; Ho et al., 1999; Shu et al., 2000; Anderson et al., 2003) might be similar to that of a semantic component (Shu and Anderson, 1997; Ho et al., 1999). However, some research has reported that knowing character pronunciation without knowing the meaning might be more common than knowing character meaning without knowing the pronunciation, and that readers were more confident in knowing character pronunciation than in knowing character meaning (Myers et al., 2007). The overall results suggest that participants performed better with phonological methods than with semantic methods.

The second reason concerns the limited number of possible character pronunciations and the relatively wide yet imprecise range of character meanings (Perfetti and Tan, 1998). There are only approximately 1,200 possible syllables in modern standard Chinese, which correspond to thousands of characters with various meanings. Nearly 88.6% of 7,263 characters listed in the *Xinhua Dictionary* (1971) have only one pronunciation, but most characters, particularly high-frequency characters, have more than one meaning (Li and Kang, 1993). The character 张, for example, has one pronunciation, *zhāng*, and eight meanings in the *Modern Chinese Dictionary* (现代汉语词典) (2016). Although learners would not know all the semantic differences, recalling one from among its various meanings might be more difficult than naming one unique pronunciation. Unlike the limited number and stability of character pronunciation, the number of semantic items for a character is comparatively difficult to define because character meanings change quickly with the emergence of new words. For instance, the original meaning of 粉 (fěn) is *powder*, yet it has acquired the new meaning of *fan* (i.e., a follower of a celebrity) in recent years. The English word *fans* has been transliterated in Chinese as 粉丝 (fěnsī), and consequently 粉 has been used as a suffix to describe the fans of popular stars, although this meaning has not been listed in Chinese dictionaries.

The third reason is related to the context-independence of character pronunciation and context-dependence of character meaning. Across different writing systems, orthographic-phonological relationships are more reliable than orthographic-semantic relationships in word identification, and word pronunciation can be retrieved without context, yet word meaning is context-dependent (Perfetti and Zhang, 1995; Perfetti and Tan, 1998). Single-pronunciation characters, constituting the majority of modern characters, are mostly pronounced the same in meaningful or non-meaningful contexts. In contrast, character meanings are highly varied and ambiguous, and difficult to define in isolation. Taking 张 (*zhāng*) for example, its pronunciation is the same across different contexts, such as 张 (zhāng), 张开 (*zhāngkāi*), 一张纸 (*yī zhāng zhǐ*), 东张西望 (*dōngzhāng xīwàng*), but the meanings are entirely different: “open” in 张开, “a measure word for paper” in 一张纸, and “look” in 东张西望. Moreover, it is difficult to judge which meaning is dominant in various meanings of a character. Even in some characters with a limited number of meanings, it is still not easy for native Chinese speakers to describe their precise meanings in isolation (Perfetti and Tan, 1998). In fact, this phenomenon relates with homophony, whereby two or more words have the same pronunciation but distinct meanings, such as *bark* and *watch* in English. Homophony is universal across different languages, and the estimated rate of homophony ranges from 3% (e.g., in Dutch) to 15% (e.g., in Japanese; Rodd et al., 2002; Ke, 2006; Trott and Bergen, 2020). Although semantic ambiguity could facilitate lexical decision to some extent, a semantic ambiguity disadvantage could interfere with word naming or tasks requiring disambiguating the meaning of ambiguous word (Borowsky and Masson, 1996; Rodd et al., 2002, 2004). Therefore, the variability of character meanings and the semantic ambiguity disadvantage might make retrieving character meaning more difficult without a context.

In sum, phonological and semantic methods focus on the phonological and semantic aspects of character recognition, respectively, and these two different methods are correlated and yet independent to some extent. In contrast, the PS method measures a person's overall knowledge of phonological and semantic properties of characters. However, whether these different methods would yield different results is still not clear.

### Measuring CSL Learners' Character Recognition

Due to the rapidly growing number of CSL learners around the world and the unique features of Chinese characters, increasing attention has been directed to CSL learners' character recognition. Chinese character acquisition has been commonly acknowledged as one of the main difficulties for CSL learners, in particular those from the non-Sinographosphere area (Allen, 2008). Measuring character recognition skills is crucial in exploring CSL learners' acquisition of Chinese characters. Different measurement methods have been utilized, and mixed results have emerged concerning the following research topics.

The first issue concerns CSL learners' comparative performance in different aspects of character recognition, particularly in character pronunciation and character meaning. Considering the complex relationship between orthography, phonology, and semantics in character recognition as discussed above, researchers have reported conflicting findings. Some studies have found that CSL learners performed better in character pronunciation than in character meaning. For instance, Li (2003) reported that both intermediate and advanced-level CSL learners in China performed better in character pronunciation than in character meaning. However, Jiang (2003) found that beginning CSL learners in China showed higher accuracy rates in character meaning than in character pronunciation. In contrast, Everson (1998) observed similar performance on these two tasks among elementary CSL learners in the United States. These inconsistent results suggest that it is still necessary to explore CSL learners' comparative achievement in character pronunciation and character meaning.

Another issue concerns the influence of individual differences on CSL learners' comparative performance in character pronunciation and character meaning, such as L2 Chinese proficiency and L1 background. It is generally accepted that CSL learners' character recognition skills improve along with their Chinese proficiency, which has been widely observed in previous research (Jiang, 2003; Li, 2003; Zhang et al., 2021).

The effect of L1 background on character acquisition refers to the phenomenon whereby CSL learners from the Sinographosphere region tend to perform better in character recognition than those from non-Sinographosphere areas. The Sinographosphere category includes CSL learners from Japan, South Korea, and Vietnam, where Chinese characters are or were commonly used in their written languages. In contrast, the non-Sinographosphere category includes CSL learners from other countries with no influence of Chinese characters on their writing systems, such as the United States, the United Kingdom, Russia, or European countries. For non-Sinographosphere learners, character learning poses a greater challenge due to the stronger orthographic contrasts between alphabetic writing systems in their L1s and the Chinese script (Chang et al., 2016a). On the other hand, CSL learners of the Sinographosphere demonstrate certain advantages in learning characters due to some shared orthographic and/or semantic features of Chinese characters in Chinese, Japanese (i.e., Kanji), and Korean (i.e., Hanja). For example, the Chinese character 剑 (jiàn, “sword”) appears as 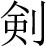 ([ken] or [tsurugi]) in Japanese and 劍 [geom] in Korean with the same meaning. Such aspects of L1 background is a crucial component of the Non-native Chinese Character Processing (NCCP) Model (Tong et al., 2015). The NCCP model includes a Chinese layer and an L1 layer, and the applicability of L1 word recognition features in Chinese character processing depends on the distance between the L1 and Chinese layers. The closer the two layers, the easier it would be to apply relevant strategies from the L1 to encode Chinese characters.

The effect of L1 background might interact with L2 Chinese proficiency in character acquisition. The achievement gap in character acquisition between Sinographosphere and non-Sinographosphere learners is assumed to decrease as CSL learners' L2 proficiency increases (Li, 2003; Zhang et al., 2021). However, it remains unclear whether the measurement method for character recognition interacts with L1 background and L2 proficiency, i.e., whether CSL learners' comparative performances in phonological, semantic, and PS methods vary across L1 background, L2 proficiency, or both. As discussed above, phonological methods are assumed to be easier than semantic methods and the PS method. However, the results summarized in Table 1 show mixed findings, as better performance in character pronunciation has not been consistently found among CSL learners with different L1 backgrounds and L2 proficiencies. Unfortunately, none of the studies in Table 1 carried out inferential statistical analysis to compare CSL learners' relative performances in these different methods. Therefore, this topic requires further exploration, which is addressed in the present study.

**Table 1:** Summary of CSL learners' performance in character pronunciation and meaning in previous studies.

**Research**	**L2 proficiency**	**L1**	**Character pronunciation**		**Character meaning**		**Comparison**
			**Task**	**Performance**	**Task**	**Performance**	
Li (2003)	Intermediate	Sinographosphere (*N* = 11)	Writing Pinyin	1,402	Forming words using target character	1,268	P > S
		Non-Sinographosphere (*N* = 13)		1,155		746	P > S
	Advanced	Sinographosphere (*N* = 7)		1,918		2,047	P < S
		Non-Sinographosphere (*N* = 11)		1,767		1,499	P > S
Jiang (2003)	Elementary	Sinographosphere (*N* = 33)	Writing Pinyin	Japan:0.95 Korea:0.98	Forming words using target character	Japan:0.98 Korea:0.95	Japan: P < S Korea: P > S
		Non-Sinographosphere (*N* = 41)		Indonesia:0.92 US:0.81		Indonesia:0.96 US:0.85	Indonesia: P < S US: P < S
Everson (1998)	Elementary	American (*N* = 20)	Online naming	26.9	Translating characters to English	26.7	P ≈ S
Xu et al. (2013)	Elementary	American (*N* = 36)	Sound-matching	R:10.00,0.88 A: 8.36,0.88 W: 8.25,0.58	Meaning-matching	R:14.94, 1.94 A: 12.94, 1.92 W: 11.11,1.44	P < S

*P, character pronunciation; S, character meaning; “>”, better than, “ < ”, worse than, “≈”, similar to. Li examined the number of characters for which participants knew pronunciations or meanings; the maximum score was 2,905. Jiang measured participants' accuracy rates in pronunciation and meaning of 30 characters and the comparative results were reanalyzed by the first author. Everson presented counts of participants' correct pronunciation and identification (measuring semantics) of 46 characters. Xu et al. examined the effects of three methods (R, reading; A, animation; W, writing) on character learning and carried out both immediate (the first number following each task) and delayed tests (the second number following each task)*.

The third issue is the effect of Chinese character recognition on L2 proficiency. Similar to the significance of word recognition for alphabetic language proficiency, as in English, the crucial role of Chinese characters in the development of Chinese language proficiency has been widely accepted. In the literature on CSL reading, character recognition has been used as an index of CSL learners' overall Chinese proficiency (Jiang and Liu, 2004; Gao, 2017; Zhang, 2018). For example, depending on the phonological task, both Wu et al. (2017) and Zhang et al. (2020) found a significant correlation (*r* = 0.45 and *r* = 0.63, respectively) between CSL learners' performance in character recognition and their Chinese proficiency level. Using a PS task, Zhang et al. (2021) reported a similar correlation coefficient between character recognition and L2 Chinese proficiency (*r* = 0.58). However, these studies used various measures for character recognition; thus, whether character recognition measured with different methods would generate similar or dissimilar effects requires further exploration.

## The Current Study

As described above, researchers have used different methods to measure Chinese character recognition, yet some research gaps remain. No study has explored the influence of certain measurement methods for character recognition on the research findings, such as the influence of L1 background and Chinese proficiency level on character recognition, and the relationship between character recognition and CSL proficiency. It is also still not clear whether the measurement methods influence the quality of Chinese character recognition tests, such as reliability, validity, and item quality. Therefore, the current study aimed to explore the following research questions.

RQ1: Does the quality (i.e., reliability, validity, and item quality) of Chinese character recognition tests differ across the phonological method, the semantic method, and the PS method?
RQ2: Do participants' performance in Chinese character recognition vary across the phonological method, the semantic method, and the PS method?
RQ3: Depending on individual differences of CSL learners in L1 background and L2 Chinese proficiency, does performance in Chinese character recognition differ across the phonological method, the semantic method, and the PS method?
RQ4: Does predictive Chinese character recognition in L2 Chinese proficiency differ across the phonological method, the semantic method, and the PS method?

## Methods

### Participants

The data of the present study were selected from a large-scale study that created a valid and reliable Chinese character size test among 318 adult CSL learners in mainland China (Zhang et al., 2021). The demographic information of the participants is summarized in Table 2. In the present study, 162 adult participants^3^ were selected based on the completeness of their background information. Their educational backgrounds varied and included 30 majors ranging from Chinese literature to electronic engineering. Participants came from 34 countries, which were further divided into two groups (i.e., Sinographosphere and non-Sinographosphere groups) according to the writing systems of their L1s.

**Table 2:** Demographic information of participants.

**Group**	**Gender**		**HSK Level**			**Age**	**CSL years**	**CSL years in China**
	**Female**	**Male**	**4**	**5**	**6**			
Pooled	111	51	60	66	36	22 (2.76)	2.76 (1.60)	1.98 (1.59)
Sinographosphere	65	19	33	33	18	22.3 (2.68)	2.72 (1.63)	1.79 (1.75)
Non-Sinographosphere	46	32	27	33	18	21.7 (2.82)	2.81 (1.58)	2.18 (1.37)

*CSL years, years of CSL learning; CSL years in China, years of CSL learning in China; Numbers in parentheses are standard deviations (SD)*.

The participants' Chinese proficiency levels were categorized based on their HSK (Hanyu Shuiping Kaoshi, “Chinese proficiency test”) performance. HSK is a standardized Chinese proficiency test for foreigners and has been widely recognized worldwide. It has six levels, with Levels 1 and 2, Levels 3 and 4, and Levels 5 and 6 representing beginner, intermediate, and advanced proficiency levels, respectively (Peng et al., 2020). Similar to IELTS and TOEFL tests, HSK scores are valid for two years from the test date. The participants were categorized into three groups according to the highest HSK level they obtained within the past two years: Level 4 (*N* = 60), Level 5 (*N* = 66), and Level 6 (*N* = 36).

### Instrument

The instrument was a Chinese character recognition test developed by the first author of this paper (see Appendix 1 for the full test), by selecting 100 Chinese characters from a pool of 3,000 characters listed in *The Graded Chinese Syllables, Characters, and Words for Teaching Chinese to the Speakers of Other Languages* (汉语国际教育用音节汉字词汇等级划分) (The State Language Affairs Commision, 2010), an official syllabus for CSL learners developed by the Confucius Institute Headquarters. The 3,000 characters in the syllabus were divided into beginning (*n* = 900), intermediate (*n* = 900) and advanced (*n* = 1,200) levels. The characters in each level were first ranked from high to low frequency and then further classified into 10 groups based on character frequency, with 300 characters in each group. The target characters were selected using stratified sampling method by further controlling the percentage of characters with single vs. multiple pronunciations, orthographic structure (e.g., top-bottom and left-right) and different degrees of phonetic regularity (e.g., regular, semiregular, and irregular). In addition, to further strengthen the representativeness of the test characters, the test characters from each group and the pooled characters in each group were comparable in the number of strokes, number of components, semantic concreteness, and morphological family size (i.e., the total number of multi-character words containing the same character in the official syllabus).

The final character recognition test included 30 beginning, 30 intermediate, and 40 advanced characters, with 95 single-pronunciation characters and five multiple-pronunciation characters. The 100 characters were printed on two pages from high to low frequency, which overlapped with character presentation according to difficulty level from easier to more difficult ones. The task involved asking the participants to write out the pronunciation in Pinyin and the meaning for a target character by forming words^4^ or translating to L1. The participants' performance in Chinese character recognition was measured *via* phonological, semantic, and PS method, respectively, whose scoring criteria were introduced below.

#### Phonological Method

The phonological method only focused on the participants' performance in character pronunciation. In scoring pronunciation, only the syllable onset and rime were considered, without considering tones. Tones were excluded for analysis mainly due to the difficulty of tone acquisition for CSL learners. For instance, Wu et al. (2006) administered a Pinyin writing task for target characters among 89 CSL learners and found that CSL learners' performance in onset and rime significantly improved along with their Chinese proficiency from beginner to intermediate level, yet their tone performance did not show such a similar growth pattern. In addition, excluding tones in scoring Chinese character recognition is commonly seen in studies involving CSL learners (Jiang, 2003; Jiang and Liu, 2004; Kim et al., 2016; Tseng et al., 2016; Kim and Shin, 2018; Xu and Maries, 2019). Tseng et al. (2016) further found that a non-tone scoring method could enhance the discrimination of test items and the validity of test results, and was more sensitive in measuring CSL learners' proficiency in character recognition. Therefore, the correct answer to the pronunciation of 你 (nǐ, “you”) could be *nǐ* (without a tone diacritic mark), *nǐ* (with the correct tone diacritic mark), or *nì* (with an incorrect tone diacritic mark). *One* point was assigned for a correct answer in pronunciation for characters with a single pronunciation or any correct answer for characters with multiple pronunciations, and zero points for an unanswered item or incorrect response. To minimize the influence of random guessing effect, scoring stopped if participants made 10 consecutive errors in pronunciation (Hung et al., 2008; Zhang and Roberts, 2019). The Cronbach alpha and McDonald's ω for the phonological method was 0.973 and 0.971, respectively.

#### Semantic Method

The semantic method only focused on the participants' performance in character meaning. The participants were encouraged to use Pinyin instead of writing in Chinese characters, because it usually takes too much time for CSL learners to write Chinese characters. In scoring the meaning, the answer was rated more holistically, and thus minor spelling or orthographic errors in Chinese characters, Pinyin, or L1 translation were ignored. Fifteen participants out of 162 participants responded to the meaning section in their L1s, such as Thai, Russian, or English, and advanced CSL learners speaking the same L1 were invited to mark these participants' responses, which was confirmed later by the first author in discussion with the graders. One point was assigned for a correct answer in meaning and zero points for unanswered items or incorrect responses; scoring stopped if participants made 10 consecutive errors in the semantic method. The Cronbach alpha and McDonald's ω for the semantic method was 0.972 and 0.971, respectively.

#### PS Method

The PS method concentrated on the participants' performance in both character pronunciation and character meaning. One point was assigned if the participants correctly responded to both the pronunciation and the meaning of the target character; otherwise, zero points were assigned. For characters with multiple pronunciations, one point was assigned only when the pronunciation and the meaning were matched. The scoring cutoff criterion in the phonological task and the semantic methods was also applied in the PS method. The Cronbach alpha and McDonald's ω for the PS method was 0.973 and 0.971, respectively.

### Procedure

This study was approved by the ethics committee by the first author's university. An informed consent form presented in Chinese was given to the participants before the test, informing them of the aim and the tasks in the study. The character test was administered in paper-and-pencil form in a group setting. It took 10–30 min for participants to complete the character test, depending on their Chinese proficiency. The participants received a gratuity or a gift for their participation. The participants were required to fill in the background questionnaire after finishing the character test. After the character test, two raters were invited to score participants' performance in the three tasks, and the correlation coefficient between the two raters' responses was 0.98, indicating high inter-rater reliability.

### Data Analysis

To answer RQ1 on the quality of character recognition test, testing analyses based on both Classical Test Theory (CTT) and the Rasch model were conducted across the three measurement methods. To answer RQ2 and RQ3, a series of ANOVA tests were carried out. Considering that the three measurement methods relied on different cognitive skills and were scored using different criteria, participants' raw scores in each measurement method were first transformed into z scores to ensure the comparability of the results yielded from the three methods. As for RQ4, a series of ordinal regression analyses were conducted to investigate how the prediction of Chinese character recognition in L2 Chinese proficiency differs across the three measurement methods, because the dependent variable of HSK level ranged from Level 4 to Level 6, and it was ordinal in nature.

## Results

The participants' performance data on the three methods are presented in Table 3.

**Table 3:** Participants' performance in measured variables.

**Group**	**HSK level**	**Measurement method**											
		**Phonological method**				**Semantic method**				**Phonological and semantic method**			
		***M***	***SD***	**MIN**	**MAX**	***M***	***SD***	**MIN**	**MAX**	***M***	***SD***	**MIN**	**MAX**
Pooled group	4	0.41	0.14	0.17	0.85	0.36	0.14	0.11	0.71	0.34	0.14	0.11	0.70
	5	0.56	0.18	0.29	0.98	0.51	0.18	0.22	0.93	0.49	0.18	0.22	0.93
	6	0.68	0.16	0.34	1.00	0.63	0.16	0.30	1.00	0.60	0.18	0.18	1.00
		0.53	0.19	0.17	1.00	0.48	0.19	0.11	1.00	0.46	0.19	0.11	1.00
Sinographosphere	4	0.40	0.10	0.17	0.85	0.35	0.11	0.12	0.71	0.33	0.10	0.11	0.70
	5	0.56	0.19	0.33	0.98	0.52	0.19	0.25	0.93	0.47	0.19	0.25	0.93
	6	0.70	0.16	0.34	1.00	0.65	0.17	0.30	1.00	0.60	0.20	0.30	1.00
		0.53	0.19	0.17	1.00	0.48	0.20	0.12	1.00	0.44	0.19	0.11	1.00
Non-Sinographosphere	4	0.43	0.18	0.21	0.72	0.37	0.17	0.11	0.67	0.36	0.18	0.11	0.67
	5	0.56	0.18	0.29	0.96	0.51	0.18	0.22	0.92	0.50	0.18	0.22	0.91
	6	0.66	0.16	0.47	1.00	0.60	0.16	0.44	1.00	0.60	0.16	0.18	1.00
		0.54	0.20	0.21	1.00	0.48	0.19	0.11	1.00	0.47	0.19	0.11	1.00

### Testing Analysis for RQ1

The main CTT-based indexes for test quality include item difficulty, item discrimination, reliability, and validity (Table 4). The general rule-of-thumb for indicating a reliable measurement was 0.20–0.80 for item difficulty and 0.20 and above for item discrimination. For item difficulty, the test with the phonological method was the easiest, followed by the one with the semantic method, and the test with the PS method was the most difficult. For item discrimination (point biserial correlation), although the three tests showed similar statistics, the test with the phonological method had more items with low discrimination than the other two methods. In addition, the three methods were similar in reliability (i.e., Cronbach alpha coefficient) and validity (i.e., correlation coefficient between accuracy rate in each measurement method and HSK level).

**Table 4:** Summary of item difficulty and item discrimination of the three measures.

**Method**	**Item difficulty**					**Item discrimination**				**Reliability**	**Validity**
	**Mean**	***SD***	**<0.20**	**0.20–0.80**	**>0.80**	**Mean**	***SD***	**<0.20**	**≥0.20**		
P	0.53	0.33	23	44	33	0.49	0.20	13	81	0.97	0.54^***^
S	0.48	0.33	28	44	28	0.49	0.16	6	92	0.97	0.54^***^
PS	0.46	0.33	32	45	23	0.49	0.17	8	92	0.97	0.51^***^

*P, phonological method; S, semantic method; PS, phonological and semantic method*.

*** *p < 0.001*.

One major shortcoming of CTT-based testing analysis is that the results are sample-dependent. Therefore, to overcome this limitation, the quality of character recognition tests using the three methods was further explored using a Rasch model *via* Winsteps software (Linacre, 2019). The quality of the test based on the Rasch model was analyzed according to person and item (Table 5). A unidimensionality analysis revealed that the three methods similarly pointed to the existence of one underlying measurement construct in character recognition, as seen in the percentage of explained variance. Next, person separation indicates the efficiency of a test in separating test-takers, and item separation indicates how well the test-takers are able to separate those items used in the test. A higher value for person separation or item separation points to a higher accuracy in measurement (Wright and Stone, 1999). It was found that the three tests were similar in person separation, person-level reliability and item-level reliability. However, the three methods differed in item separation, in which the value of the PS method was the highest, followed by that of the semantic method, and that of the phonological method was the lowest. Finally, the rule-of-thumb for interpreting parameter-level mean-square fit statistics was 0.50–1.5 as productive for measurement, and 1.5–2.0 as unproductive for measurement construction.^5^ Therefore, a character recognition test with the semantic method might be productive for measurement, and tests with the other two methods might be unproductive for measurement.

**Table 5:** Summary of the person separation and item separation values.

**Method**	**% of variance explained**	**Person**				**Item**			
		**SEP**	**REL**	**IMNSP**	**OMNSP**	**SEP**	**REL**	**IMNSP**	**OMNSP**
P	65.2%	5.56	0.97	0.97	1.28	6.30	0.98	0.98	1.71
S	63.6%	5.51	0.97	0.98	1.21	7.57	0.98	0.98	1.36
PS	64.6%	5.57	0.97	0.97	1.35	8.99	0.99	0.98	1.60

*P, phonological method; S, semantic method; PS, phonological and semantic method; SEP, separation; REL, reliability; IMNSP, infit mean-square, an inlier-pattern-sensitive fit statistic; OMNSP, outfit mean-square, an outlier-sensitive fit statistic*.

### ANOVA Tests for RQ2

As mentioned in the method section, the participants' performance in the PS method was dependent on those in the other two methods, which violated the assumption of ANOVA tests. To overcome this limitation, the participants were systematically divided into three groups by mainly controlling for the participants' background variables and Chinese language proficiency (Table 6). The three groups did not differ significantly from each other in accuracy rates for each of the three measurement methods. Thus, the three groups could be seen as paired and matched in Chinese recognition skills. The participants' performance in each of the three measurement methods was randomly selected from each of the three groups and used for ANOVA tests. In the final analysis, the accuracy rates in the phonological method from Group 1, the semantic method from Group 2 and the PS method from Group 3 were chosen for between-subjects ANOVA tests, with accuracy rate as the dependent variable and measurement method as the independent variable. The results showed that the main effect of the measurement method was not significant, and that the effect size was small: *F*_(2, 159)_ = 1.48, η^2^ = 0.02, and ω^2^ = 0.01.

**Table 6:** Summary of the three randomly selected groups.

**Variable**			**Group 1**	**Group 2**	**Group 3**
L1	Sinographosphere	HSK4	11	11	11
		HSK5	11	11	11
		HSK6	6	6	6
	Non-Sinographosphere	HSK4	9	9	9
		HSK5	11	11	11
		HSK6	6	6	6
Age		Mean (*SD*)	21.8 (2.52)	22.3 (3.66)	21.9 (1.78)
		ANOVA	*F*_(2, 159)_ = 0.60, *p* = 0.55, η^2^ = 0.007		
Years of CSL learning		Mean (*SD*)	2.53 (1.21)	2.92 (1.83)	2.84 (1.69)
		ANOVA	*F*_(2, 159)_ = 0.91, *p* = 0.41, η^2^ = 0.01		
Phonological method		Mean (*SD*)	0.53(0.18)	0.52 (0.19)	0.54 (0.22)
		ANOVA	*F*_(2, 159)_ = 0.23, *p* = 0.80, η^2^ = 0.003		
Semantic method		Mean (*SD*)	0.48 (0.17)	0.47 (0.20)	0.50 (0.21)
		ANOVA	*F*_(2, 159)_ = 0.21, *p* = 0.81, η^2^ = 0.003		
Phonological and semantic method		Mean (*SD*)	0.46 (0.17)	0.44 (0.19)	0.47 (0.22)
		ANOVA	*F*_(2, 159)_ = 0.26, *p* = 0.77, η^2^ = 0.003		

### ANOVA Tests for RQ3

To answer RQ3, a series of two-way ANOVA tests were carried out, with accuracy rate as the dependent variable and L1 background (Sinographosphere vs. non-Sinographosphere) and Chinese proficiency (HSK Level 4 vs. Level 5 vs. Level 6) as the independent variables. As seen in Table 7, the results of the two-way ANOVA tests were similar across the three measurement methods: the main effect of L1 background and the interaction effect between L1 background and HSK level on character recognition was insignificant, and the effect size was very similarly small (Cohen, 1988, 0–0.06 for small, 0.06–0.14 for medium, and a number >0.14 for large); the main effect of HSK level on character recognition was significant and the effect size was similarly large.

**Table 7:** Summary of ANOVA tests for each measurement method.

**Method**	**L1**	**HSK level**	L1 *HSK level
Phonological	*F*_(1, 156)_ = 0.03, *p =* 0.86, η^2^ = 0, ω^2^ = 0	*F*_(2, 156)_ = 31.70, *p* < 0.001, η^2^ = 0.29, ω^2^ = 0.28	*F*_(2, 156)_ = 0.49, *p =* 0.61, η^2^ = 0.004, ω^2^ = 0
Semantic	*F*_(1, 156)_ = 0.21, *p =* 0.65, η^2^ = 0.001, ω^2^ = 0	*F*_(2, 156)_ = 31.64, *p* < 0.001, η^2^ = 0.29, ω^2^ = 0.28	*F*_(2, 156)_ = 0.39, *p =* 0.68, η^2^ = 0.004, ω^2^ = 0
Phonological and semantic	*F*_(1, 156)_ = 0.47, *p =* 0.49, η^2^ = 0.002, ω^2^ = 0	*F*_(2, 156)_ = 27.81, *p* < 0.001, η^2^ = 0.26, ω^2^ = 0.25	*F*_(2, 156)_ = 0.12, *p =* 0.88, η^2^ = 0.001, ω^2^ = 0

### Regression Analysis for RQ4

A set of ordinal regression tests were carried out to answer RQ4. L2 Chinese proficiency was used as the dependent variable due to following reasons. Although researchers have commonly regarded Chinese character recognition skill as an indicator of L2 Chinese proficiency (Zhang, 2018; Zhang et al., 2020), they used different tasks to measure character recognition and have not reached a consensus about which method could best tap character recognition skill. Also, Chinese character recognition skill is an integrated component of, but does not equate to, Chinese proficiency. According to theories concerning second language proficiency (Bachman, 1990; Hulstijn, 2012), Chinese proficiency represents an individual's global performance in various language elements (e.g., characters and grammar) and language skills (e.g., reading and listening). Recent research further found that using character recognition skill as a measure of L2 Chinese proficiency was less powerful than other comprehensive measures such as HSK test (Zhang et al., 2020). Therefore, it is reasonable to add L2 Chinese proficiency as the dependent variable predicted by Chinese character recognition.

The correlation matrix between the measured variables is presented in Table 8, where one can see that participants' standardized scores in the three measurement methods were highly correlated. However, since researchers have not reached a consensus on the optimal method to measure character recognition, and since the correlation coefficients varied across the Sinographosphere and non-Sinographosphere groups (Table 8), exploring the predictive power of these three methods in L2 Chinese proficiency could deepen our understanding of the impact of different methods on research findings across CSL learners with different L1 backgrounds. Therefore, three-step hierarchical regression tests were administered (Tables 9, 10). In the first step, a base model was created for the pooled participants, and the predictors included individual learner variables such as age, gender, and L1. A second-step model was created by adding years of CSL learning. In the third step, participants' accuracy rates in each of the three methods were added separately^6^. Similarly, a series of regression tests excluding L1 from the predictor variables were conducted in the Sinographosphere and the non-Sinographosphere group, respectively.

**Table 8:** Correlation matrix between measured variables.

**Pooled group**	**1**	**2**	**3**	**4**	**5**
1. CSL years	—				
2. HSK level	0.56^***^	—			
3. P	0.56^***^	0.54^***^	—		
4. S	0.51^***^	0.54^***^	0.94^***^	—	
5. PS	0.49^***^	0.51^***^	0.89^***^	0.93^***^	—
**Sinographosphere group**					
1. CSL years	—				
2. HSK level	0.43^***^	—			
3. P	0.69^***^	0.61^***^	—		
4. S	0.64^***^	0.59^***^	0.95^***^	—	
5. PS	0.60^***^	0.55^***^	0.84^***^	0.88^***^	—
**Non-Sinographosphere group**					
1. CSL years	—				
2. HSK level	0.70^***^	—			
3. P	0.41^***^	0.46^***^	—		
4. S	0.35^***^	0.47^***^	0.93^***^	—	
5. PS	0.37^***^	0.48^***^	0.94^***^	0.99^***^	—

*** *p < 0.001*.

*CHL learner, learning Chinese as a heritage language; CSL years, years of CSL learning; CSL years in China, years of CSL learning in China; HSK level, highest level of the HSK test that participants passed; P, phonological method; S, semantic method; PS, phonological and semantic method*.

**Table 9:** Summary of results for ordinal regression tests.

**Group**	**Step**	**Model**	**AIC**	**BIC**	** ${\text{R}}_{\text{MCF}}^{2}$ **	${\text{R}}_{\text{MCF}}^{2}$ change*	** ${\text{R}}_{\text{CS}}^{2}$ **	${\text{R}}_{\text{CS}}^{2}$ change*	**χ^2^**	**df**	***p***
Pooled group	1	HSKlevel~age+gender+L1	353	369	0.001		0.001		2.73	3	0.44
	2	HSKlevel~age+gender+L1+CSLyrs	293	311	0.19	0.189	0.13	0.129	65.4	4	<0.001
	3a	HSKlevel~age+gender+L1+CSLyrs+P	279	301	0.23	0.04	0.15	0.02	80.8	5	<0.001
	3b	**HSKlevel** **~** **age+gender+L1+CSLyrs+S**	**274**	**296**	**0.25**	**0.06**	**0.16**	**0.03**	**85.7**	**5**	**<0.001**
	3c	HSKlevel~age+gender+L1+CSLyrs+PS	276	298	0.24	0.05	0.16	0.03	83.9	5	<0.001
Sinographo-sphere	1	HSKlevel~age+gender	184	194	0.01		0.01		2.58	2	0.28
group	2	HSKlevel~age+gender+CSLyrs	171	183	0.10	0.09	0.07	0.06	17.5	3	<0.001
	3a	**HSKlevel** **~** **age+gender+CSLyrs+P**	**155**	**170**	**0.20**	**0.10**	**0.13**	**0.06**	**35.4**	**4**	**<0.001**
	3b	HSKlevel~age+gender+CSLyrs+S	157	171	0.19	0.09	0.13	0.06	34.3	4	<0.001
	3c	HSKlevel~age+gender+CSLyrs+PS	160	175	0.17	0.07	0.11	0.04	30.6	4	<0.001
Non-Sinographo-sphere	1	HSKlevel~age+gender	165	174	0.06		04		10.3	2	0.01
group	2	HSKlevel~age+gender+CSLyrs	120	132	0.34	0.28	0.22	0.18	56.7	3	<0.001
	3a	HSKlevel~age+gender+CSLyrs+P	118	132	0.36	0.02	0.23	0.01	60.6	4	<0.001
	3b	**HSKlevel** **~** **age+gender+CSLyrs+S**	**114**	**128**	**0.39**	**0.05**	**0.24**	**0.02**	**64.5**	**4**	**<0.001**
	3c	HSKlevel~age+gender+CSLyrs+PS	115	129	0.38	0.04	0.24	0.02	64	4	<0.001

1. ^*^${\text{R}}_{\text{McF}}^{2}$ change is the difference between the value of ${\text{R}}_{\text{McF}}^{2}$ of the first-step model and the second-step model in each group.

*${\text{R}}_{\text{CS}}^{2}$ change is the difference between the value of ${\text{R}}_{\text{CS}}^{2}$ of the first-step model and the second-step model in each group*.

*${\text{R}}_{\text{McF}}^{2}$ change and ${\text{R}}_{\text{CS}}^{2}$ change indicate the percentage of variance explained by the predictor variables*.

*2. HSKlevel, the highest level of HSK test participants obtained; CSLyrs, years of CSL learning; P, phonological method; S, semantic method; PS, phonological and semantic method*.

*3. AIC (Akaike information criterion) and BIC (Bayesian information criterion) are criteria for model selection. Models with lower AIC or lower BIC are preferred*.

*4. The best model for each group was in bold*.

**Table 10:** Summary of the predictive power of three methods in Chinese language proficiency.

**Group**	**Predictor**	**Estimate**	**SE**	***p***	**Odds ratio**	**Estimate**	**SE**	***p***	**Odds ratio**	**Estimate**	**SE**	***p***	**Odds ratio**
Pooled group	Age	0.07	0.06	0.23	1.07	0.08	0.06	0.18	1.08	0.08	0.06	0.11	1.09
	L1	−0.10	0.34	0.77	0.91	−0.13	0.34	0.71	0.88	−0.04	0.34	0.92	1.04
	Gender	−0.35	0.39	0.38	0.71	−0.41	0.40	0.30	0.66	−0.35	0.40	0.37	0.70
	CSL years	0.70	0.15	<0.001	2.02	0.74	0.15	<0.001	2.10	0.78	0.15	<0.001	2.18
	P	4.05	1.06	<0.001	57.52								
	**S**					**4.49**	**1.04**	**<0.001**	**89.32**				
	PS									4.28	1.03	<0.001	72.05
Sinographo-sphere group	Age	−0.01	0.09	0.89	0.99	−0.005	0.09	0.96	0.99	0.01	0.09	0.93	1.01
	Gender	0.01	0.57	0.98	1.02	0.02	0.58	0.97	1.02	0.08	0.59	0.90	1.08
	CSL years	0.06	0.21	0.78	1.06	0.19	0.20	0.35	1.21	0.32	0.21	0.13	1.37
	**P**	**7.02**	**1.78**	**<0.001**	**1122.18**								
	S					6.19	1.61	<0.001	488.45				
	PS									5.30	1.56	<0.001	200.97
Non-sinographo-sphere group	Age	0.10	0.10	0.27	1.11	0.11	0.09	0.22	1.12	0.11	0.09	0.22	1.12
	Gender	−0.48	0.56	0.39	0.62	−0.58	0.57	0.31	0.56	−0.59	0.57	0.30	0.55
	CSL years	1.26	0.26	<0.001	3.51	1.28	0.26	<0.001	3.58	1.27	0.26	<0.001	3.55
	P	2.91	1.50	0.05	18.32								
	**S**					**4.11**	**1.52**	**0.01**	**60.95**				
	PS									3.97	1.52	0.01	52.95

*CSLyrs, years of CSL learning; P, phonological method; S, semantic method; PS, phonological and semantic method*.

*The strongest predictor for each group was in bold*.

Different types of pseudo *R*^2^ indices have been used in logistic regression, and $R_{\text{MCF}}^{2}$ proposed by McFadden (1974) and $R_{\text{CS}}^{2}$ proposed by Cox and Snell (1989) are commonly recommended (Smith and McKenna, 2012). Therefore, these two pseudo *R*^2^ indices are presented here. The percentage of variance by HSK level explained by each measurement method was similar in each group^7^, and the effect sizes were small (Cohen, 1988; Hair et al., 2011). For $R_{\text{McF}}^{2}$ change, the percentage of variance explained by each method was 0.04–0.06 in the pooled group, 0.07–0.10 in the Sinographosphere group, and 0.02–0.05 in the non-Sinographosphere group. For $R_{\text{CS}}^{2}$ change, the percentage of variance explained by each method was 0.02–0.03 in the pooled group, 0.04–0.06 in the Sinographosphere group, and 0.01–0.02 in the non-Sinographosphere group. That is, the differences in the contributions of character recognition to L2 Chinese proficiency across the three methods were very small. Table 10 shows the predictive power of each method for L2 Chinese proficiency. It can be seen that, based on the odds ratio values, the most robust method of Chinese character recognition in predicting HSK level was the semantic method in the pooled group and the non-Sinographosphere group, and the phonological method in the Sinographosphere group.

Altogether, according to the AIC and BIC values (the smaller the better), and $R_{\text{McF}}^{2}$, $R_{\text{CS}}^{2}$ and the odds ratios (the larger the better), the semantic method seems to yield the best model fit in the pooled and non-Sinographosphere group, and the phonological method seems to generate the best model fit in the Sinographosphere group.

## Discussion

The present study explored whether three different measurement methods (phonological, semantic, and phonological plus semantic) for Chinese character recognition would lead to different results. The overall findings from 162 CSL learners' data revealed that these three methods produced both similarities and differences in results for Chinese character recognition. In terms of similarities, the participants' performance in character recognition was similar across the three measurement methods; the influence of L1 background and Chinese proficiency level on character recognition did not vary across the measurement type; and the contribution of character recognition to L2 Chinese proficiency as measured by each measurement method was similar. Yet some differences were found, in that the three methods differed in the quality of the character recognition test, and different methods yielded the best model and showed different predictions for L2 Chinese proficiency across different L1 backgrounds.

### Similarities in Results of the Three Measurement Tasks

The similarities among the three measurement tasks for character recognition in this study need to be accounted for. The results for RQ2 suggest that the participants' performance in Chinese character recognition might be similar across the three methods. The results for RQ3 indicate that the three methods might have comparable power to differentiate CSL learners with variations in L1 background and HSK level. The results for RQ4 found that the percentage of variance in L2 Chinese proficiency explained by Chinese character recognition was similar across the three measurement methods, suggesting that each method might make a similar contribution to L2 Chinese proficiency. The overall results might be explained from both theoretical and pedagogical perspectives.

From a theoretical perspective, these results support the interconnection between orthographic, phonological, and semantic representations of word recognition (Seidenberg and McClelland, 1989; Plaut and Booth, 2000; Lupker, 2005; Perfetti et al., 2005) and Chinese character recognition (Chang et al., 2016b; Reichle and Yu, 2018) to some extent. The results might be also explained by the finding that that brain areas such as the left middle frontal gyrus, the left superior parietal lobule, and the left mid-fusiform gyrus are activated in both phonological and semantic processing of characters (Wu et al., 2012). That is, the phonological, semantic, and orthographic information might be activated in an interconnected manner during the process of Chinese character recognition for CSL learners, which further leads to the participants' comparable performance in the three measurement methods.

From a pedagogical perspective, the similarities in the three measurement methods may stem from how characters are introduced, taught, and tested among CSL learners. The orthography, pronunciation, and meaning of Chinese characters are almost always taught together to adult CSL learners. In CSL textbooks, a typical procedure for introducing new characters is first presenting orthographic forms, followed by pronunciation (sometimes pronunciations are written above the characters), and finally, meaning. In general, Chinese tests examine students' knowledge of character orthography, phonology, and meaning in a comprehensive manner by requiring students to provide pronunciations and meanings for target characters or to write characters based on given pronunciations and meanings. That is, CSL learners are trained to achieve balanced performance in orthography, phonology, and meaning. In addition, character recognition has been validated as a unidimensional psychological construct (Wen et al., 2016), suggesting that orthography, phonology, and meaning might be three interconnected components in recognizing Chinese characters.

### Differences in Results of the Three Measurement Methods

The first difference relates to the quality of character recognition test, which differed across the three methods. The overall results of CTT- and Rasch-based testing analysis indicate that the semantic method could be considered an optimal one for generating a character recognition test with higher quality. One possible reason could be the difficulty of the three measurement methods. As discussed above, the difficulty of the semantic method falls between that of the phonological method and that of the PS method. However, the semantic method and the PS method were more similar in their results from the test analysis (Tables 4, 5). For instance, the two methods had a comparable number of items with low discrimination and a similar value in item separation. That is, the semantic method could enhance the quality of character recognition test to some extent, which might relate with the internal characteristics of Chinese characters, such as the relative difficulty of correctly retrieving character meaning from semantic radicals, and the context-dependence and semantic ambiguity of character meanings (Perfetti and Zhang, 1995; Perfetti and Tan, 1998; Myers et al., 2007). However, due to the limited sample size in the present study, more studies are needed to explore how the measurement method could influence the quality of character recognition tests.

The second difference concerns the different patterns found in the best model fits for L2 proficiency in the different L1 groups. The method that might yield the best model fits and the strongest prediction for L2 Chinese proficiency was the phonological method for the Sinographosphere group and the semantic method for the pooled group and non-Sinographospheric group. This suggests that L1 background might influence the relationship between the components of character recognition and L2 Chinese proficiency.

For Sinographosphere learners, the phonological aspect of character recognition might be crucial for their Chinese proficiency, arguably due to the great differences in character pronunciations between Chinese and their L1s. Japanese Kanji and Korean Hanja share more similarities with Chinese characters in meaning than in phonology (Daniels and Bright, 1996; Kuriya, 2004), as the pronunciations of Kanji^8^ and Hanja, which were borrowed from pre-modern Chinese languages, are very different from their modern Chinese pronunciations (Chen, 1999; Sun, 2006). The orthographic features of Chinese characters (i.e., simplified characters), Kanji (i.e., reformed characters within Japanese), and Hanja (i.e., traditional characters) are also still somewhat different, but the meanings of characters remain more similar or have changed more slowly than their orthography and pronunciation. In general, Sinographosphere learners already have some knowledge of character meanings in their L1s, due to exposure to Kanji or Hanja in daily life and/or explicit teaching of Kanji or Hanja in schools, so remembering the meanings of Chinese characters would be easier than recalling pronunciations for CSL learners from the Sinographosphere (Chen, 2001; Liu, 2013). As a result, their knowledge of Chinese character pronunciations could be the best predictor for Chinese proficiency.

In contrast, for non-Sinographosphere learners who have not been exposed to meaning-based Chinese characters before learning Chinese, knowledge of character meaning might be more important for their Chinese proficiency, owing to the internal characteristics of Chinese characters, such as the complexity and difficulty of character semantics compared to character pronunciation. The probability of guessing a correct or an approximate character pronunciation is higher than for predicting character meaning (Perfetti and Zhang, 1995; Perfetti and Tan, 1998; Myers et al., 2007), so meaning is more difficult to learn or master than pronunciation. Research has also found that semantic radical information that is opaque or unknown to learners tends to generate more errors in reading or identifying characters than other components (Peng, 1982). Therefore, probably because of these internal characteristics of the script, character meaning could be a more challenging yet crucial factor in learning than pronunciation. Also, for CSL learners who are experienced only with phonologically based L1 writing systems without any previous exposure to Chinese characters such as the non-Sinographosphere learners in this study, features by which Chinese characters can convey some hint of meaning would be more marked or outstanding than pronunciation cues (Yu and Bellassen, 2021). It is likely that the drastic contrast in writing systems between L1 and Chinese may draw CSL learners' more immediate attention to meaning than pronunciation. This explanation is in line with American CFL learners' bias for semantic strategies in lexical decision tasks (Williams and Bever, 2010).

The differences found in the Sinographosphere and non-Sinographosphere groups are consistent with the literature on L1 influence in L2 reading. It has been commonly found that L2 learners' performance in word recognition might be influenced by the characteristics of their L1s. For example, Chinese or Japanese ESL learners tend to depend on orthographic strategies in recognizing English words, yet ESL learners using alphabetic L1s are likely to rely on phonological strategies (Brown and Haynes, 1985; Wang et al., 2003; Koda, 2008; Zhao et al., 2017). Similar results were found for character recognition among CSL learners. Knowledge of character pronunciation highly correlated with knowledge of meaning among non-Sinographospheric learners, but not among Sinographosphere learners (Jiang, 2003). In addition, phonological awareness, rather than phonetic radical awareness, significantly predicted character reading and writing among English and Arabic CSL learners (Zhang and Roberts, 2019). These overall results point to the universal influence of L1 on L2 reading across L2 learners of different target languages.

### Implications

To the best of our knowledge, this is the first empirical study to compare these three typical measurement methods of character recognition. The findings have theoretical and practical significance.

From a theoretical perspective, the current study provides evidence for some longstanding issues in literacy acquisition. The close correlations between the phonological, semantic, and PS methods, along with the similar contributions of each measurement method to L2 Chinese proficiency, might validate the close relationship between phonological and semantic processing in character recognition (Perfetti and Zhang, 1995; Perfetti and Tan, 1998; Zhou et al., 1999) and the interaction between phonology and meaning in word recognition (Seidenberg and McClelland, 1989; Plaut and Booth, 2000; Lupker, 2005).

Moreover, our findings can offer new insights into the role of L1 background in L2 literacy acquisition. The different patterns in the best model fits for the phonological and semantic methods for predicting Chinese proficiency across different L1 groups point to the effect of L1 orthographic background on the relationship between the different components of word recognition and L2 proficiency. This finding extends the influence of L1 background on L2 acquisition from individual components such as processing strategies in word recognition (Brown and Haynes, 1985; Wang et al., 2003; Koda, 2008; Zhao et al., 2017) to relationships between the components of lexical learning and holistic language proficiency. Therefore, further research on the effects of L1 influence on L2 learning is suggested, which might be helpful in providing a clearer picture of the role of L1 background in the process of acquiring an L2.

On a practical level, the results of the current study have two implications. The findings validate the interchangeability of the three methods in measuring character recognition to some extent, and provide empirical evidence for commonly used phonological methods in existing studies. This also has certain implications for using character recognition as an index of L2 Chinese proficiency for research purposes (Zhang, 2018; Zhang et al., 2020). Although the three measurement methods could be interchangeable in some cases, it is advised that the prime measurement of character recognition be selected according to the participants' L1 background. Based on the results of testing analysis and character acquisition in the present study, phonological tasks are recommended for Sinographosphere learners, and semantic tasks are recommended for non-Sinographosphere learners and participants with mixed L1 backgrounds.

## Conclusion

The current study examined three different methods of measuring character recognition among 162 CSL learners. The results suggest that the three measurement methods could lead to both similar and differing results, and that selecting an appropriate method for character recognition abilities is important. This study has theoretical and practical significance for research involving CSL participants' Chinese character recognition. In addition, this study's findings can deepen our understanding of the relationship between phonology and semantics of Chinese characters, as well as the influence of L1 background on L2 acquisition, and provide clearer guidance on the preferred instruments for measuring Chinese character recognition in the future.

For future studies, the following two points can be considered for improvement. The current study was conducted in an L2 learning setting, where Chinese is the official, dominant language, not in a foreign language learning context. Thus, it is still not clear whether the findings of this study could be generalized to other groups, such as Chinese learners outside China or even L1 Chinese speakers. Also, the task of the current study was writing pronunciations in Pinyin and meanings of target characters in only short-answer question format. Other task types, such as a multiple-choice question format, verbal responses (e.g., reading aloud) or a cloze test, might yield dissimilar results.

## Data Availability Statement

The original contributions presented in the study are included in the article/Supplementary Materials, further inquiries can be directed to the corresponding author.

## Ethics Statement

The studies involving human participants were reviewed and approved by College of International Education, Minzu University of China. The patients/participants provided their written informed consent to participate in this study.

## Author Contributions

HZ was responsible for research design, data collection, and article drafting. SK was responsible for research design, data analysis, and article drafting. XZ was responsible for research design and data collection. All authors contributed to the article and approved the submitted version.

## Funding

This study was funded by Ministry of Education of China (20YJC740088) and Beijing Social Science Foundation (17YYC018).

## Conflict of Interest

The authors declare that the research was conducted in the absence of any commercial or financial relationships that could be construed as a potential conflict of interest.

## Publisher's Note

All claims expressed in this article are solely those of the authors and do not necessarily represent those of their affiliated organizations, or those of the publisher, the editors and the reviewers. Any product that may be evaluated in this article, or claim that may be made by its manufacturer, is not guaranteed or endorsed by the publisher.

## Footnotes

^1^The terms of *phonology* and *semantics* are from Seidenberg and McClelland's word reading model based on the three components of words: *orthography* (spelling), *phonology* (pronunciation), and *semantics* (meaning; Seidenberg and McClelland, 1989).

^2^In this paper, *Sinographosphere* refers to the “Chinese character cultural sphere (汉字文化圈),” such as Korea, Japan, or Vietnam, following Handel (2019).

^3^Twenty-eight participants of 162 participants reported that they learned Chinese as a heritage language (CHL), but what they learned mainly focused on spoken Chinese, not written Chinese, and they spoke it mainly at home to communicate with their family members. Since this study focuses on Chinese characters and literacy skills, we included them in this study.

^4^Forming words or phrases using a target character is a commonly used method to measure participants' knowledge of Chinese character meanings. The meanings of some characters might not be translatable into CSL learners' L1, or some characters cannot be used as independent morphemes; for instance, neither character in 蝴蝶 (húdié, “butterfly”) can be used independently. Thus, forming words or phrases could provide a context where the meaning of a character could be determined. It is possible that CSL learners can form a word or phrase without knowing the meaning of a character, but this situation is commonly observed mostly among beginning CSL learners. Considering that the Chinese proficiency of the participants in the present study were intermediate and above, it is safe to assume that this method would not significantly skew the results.

^5^ https://www.winsteps.com/winman/misfitdiagnosis.htm

^6^The participants' performance in the three method were not added to a single regression model, because the three variables were highly correlated. In the regression model [HSK level ~ age + gender + years of CSL learning + P (phonological method) + S (semantic method) + PS (phonological and semantic method)], where HSK level was considered a continuous variable, the VIF value of the P method, the S method, and the PS method was 9.75, 14.96, and 7.92, respectively, higher than the threshold value of 10, suggesting a high degree of multi-collinearity.

^7^The percentage of variance in Chinese proficiency explained by each assessment method was calculated by the value of $R_{\text{McF}}^{2}$ change or $R_{\text{CS}}^{2}$ change from the 2nd-step model to the 3rd-step model.

^8^Kanji have *on* and *kun* readings. The *on* reading is the original pronunciation of Chinese characters, while *kun* reading uses Japanese native words whose meaning are equivalent to Chinese characters.
