# Supplementary material for: A Comparative Study of Three Measurement Methods of Chinese Character Recognition for L2 Chinese Learners
Source: Front Psychol. 2022 Mar 25;13:753913. doi: 10.3389/fpsyg.2022.753913 (PMC8990832; doi:10.3389/fpsyg.2022.753913)
Supplement: Supplementary file 1 [file Table_1.docx]

Appendix: Hanzi recognition size test for learners of Chinese as a second language

**留学生识字量测试**

Hanzi recognition size test for learners of Chinese as a second language

请按顺序写出下列汉字的拼音和意义（组词、造句或翻译，可用拼音）。如果连续有10个不知道读音或意义，请停止。感谢您的帮助!

Please write down Pinyin and meaning of the following Hanzi. You can use words or phrases or translation for the meaning. Please stop if you don’t know the pronunciation or meaning of 10 characters in a row. Thanks for your help.

例子： Hanzi Pinyin Yisi

你 nǐ ( nihao/~hao/~好/you )

|  | Hanzi | Pinyin | Yisi |  |  | Hanzi | Pinyin | Yisi |
| --- | --- | --- | --- | --- | --- | --- | --- | --- |
| 1 | 作 |  | （ ） |  | 26 | 款 |  | （ ） |
| 2 | 心 |  | （ ） |  | 27 | 茶 |  | （ ） |
| 3 | 部 |  | （ ） |  | 28 | 网 |  | （ ） |
| 4 | 开 |  | （ ） |  | 29 | 祝 |  | （ ） |
| 5 | 情 |  | （ ） |  | 30 | 联 |  | （ ） |
| 6 | 最 |  | （ ） |  | 31 | 阶 |  | （ ） |
| 7 | 或 |  | （ ） |  | 32 | 党 |  | （ ） |
| 8 | 公 |  | （ ） |  | 33 | 县 |  | （ ） |
| 9 | 必 |  | （ ） |  | 34 | 妇 |  | （ ） |
| 10 | 农 |  | （ ） |  | 35 | 圆 |  | （ ） |
| 11 | 强 |  | （ ） |  | 36 | 毒 |  | （ ） |
| 12 | 花 |  | （ ） |  | 37 | 献 |  | （ ） |
| 13 | 连 |  | （ ） |  | 38 | 哲 |  | （ ） |
| 14 | 乐 |  | （ ） |  | 39 | 鬼 |  | （ ） |
| 15 | 效 |  | （ ） |  | 40 | 妻 |  | （ ） |
| 16 | 城 |  | （ ） |  | 41 | 汗 |  | （ ） |
| 17 | 拉 |  | （ ） |  | 42 | 煤 |  | （ ） |
| 18 | 势 |  | （ ） |  | 43 | 潜 |  | （ ） |
| 19 | 围 |  | （ ） |  | 44 | 震 |  | （ ） |
| 20 | 议 |  | （ ） |  | 45 | 贡 |  | （ ） |
| 21 | 突 |  | （ ） |  | 46 | 艰 |  | （ ） |
| 22 | 富 |  | （ ） |  | 47 | 融 |  | （ ） |
| 23 | 模 |  | （ ） |  | 48 | 肩 |  | （ ） |
| 24 | 错 |  | （ ） |  | 49 | 贴 |  | （ ） |
| 25 | 男 |  | （ ） |  | 50 | 兼 |  | （ ） |
|  | Hanzi | Pinyin | Yisi |  |  | Hanzi | Pinyin | Yisi |
| 51 | 裁 |  | （ ） |  | 76 | 溃 |  | （ ） |
| 52 | 闲 |  | （ ） |  | 77 | 倘 |  | （ ） |
| 53 | 桃 |  | （ ） |  | 78 | 衍 |  | （ ） |
| 54 | 牢 |  | （ ） |  | 79 | 髓 |  | （ ） |
| 55 | 寺 |  | （ ） |  | 80 | 鸽 |  | （ ） |
| 56 | 仓 |  | （ ） |  | 81 | 僵 |  | （ ） |
| 57 | 扇 |  | （ ） |  | 82 | 膊 |  | （ ） |
| 58 | 嘉 |  | （ ） |  | 83 | 爵 |  | （ ） |
| 59 | 饼 |  | （ ） |  | 84 | 挪 |  | （ ） |
| 60 | 傲 |  | （ ） |  | 85 | 匙 |  | （ ） |
| 61 | 脉 |  | （ ） |  | 86 | 羡 |  | （ ） |
| 62 | 岩 |  | （ ） |  | 87 | 兑 |  | （ ） |
| 63 | 荡 |  | （ ） |  | 88 | 拧 |  | （ ） |
| 64 | 腹 |  | （ ） |  | 89 | 删 |  | （ ） |
| 65 | 芽 |  | （ ） |  | 90 | 惦 |  | （ ） |
| 66 | 饲 |  | （ ） |  | 91 | 秦 |  | （ ） |
| 67 | 膀 |  | （ ） |  | 92 | 藻 |  | （ ） |
| 68 | 斥 |  | （ ） |  | 93 | 蚀 |  | （ ） |
| 69 | 胎 |  | （ ） |  | 94 | 巫 |  | （ ） |
| 70 | 鹿 |  | （ ） |  | 95 | 绷 |  | （ ） |
| 71 | 旬 |  | （ ） |  | 96 | 菩 |  | （ ） |
| 72 | 巢 |  | （ ） |  | 97 | 颓 |  | （ ） |
| 73 | 譬 |  | （ ） |  | 98 | 汛 |  | （ ） |
| 74 | 契 |  | （ ） |  | 99 | 嫦 |  | （ ） |
| 75 | 凳 |  | （ ） |  | 100 | 诽 |  | （ ） |
